# Detection of multidrug-resistant *Salmonella enterica* subsp. *enterica* serovar Typhi isolated from Iraqi subjects

**DOI:** 10.14202/vetworld.2021.1922-1928

**Published:** 2021-07-26

**Authors:** Hamzah Abdulrahman Salman, Ali Mohammed Abdulmohsen, Mays Noori Falih, Zahraa Mohmoud Romi

**Affiliations:** 1 Department of Medical Laboratory Techniques, College of Medical Sciences Techniques, The University of Mashreq, Baghdad, Iraq; 2 The Biological Society of Iraq, Baghdad, Iraq

**Keywords:** antibiotics susceptibility, enteric fever, multidrug-resistant, *Salmonella*, typhoid fever, Vitek 2 compact

## Abstract

**Background and Aim:**

Enteric fever initiated by *Salmonella enterica* subsp. *enterica* serovar Typhi (*S*. Typhi) is among the most consistent disease worldwide, particularly in developing countries. The present study aimed to isolate and identify *S*. Typhi from typhoid suspected patients and determine their antibacterial susceptibility testing.

**Materials and Methods:**

Thirty blood samples were collected from typhoid suspected patients in Baghdad, Iraq. The samples were cultured on SS agar and XLD agar for screening of *S*. Typhi. The suspected colonies were picked up and subjected to Vitek 2 compact for biochemical identification and antibacterial susceptibility testing of the organisms. Molecular identification of the isolates was performed by real-time polymerase chain reaction (RT-PCR).

**Results:**

Black colonies were observed on cultured plates. Out of 30 samples, 27 and 29 isolates were identified as *S*. Typhi using Vitek 2 compact and RT-PCR, respectively. The data of the present study revealed that the strains of *S*. Typhi were showing multidrug resistance. All *S*. Typhi strains exhibited resistance to penicillins (ticarcillin and piperacillin), cephalosporins 4^th^ G (cefepime), and monobactam (aztreonam). However, all the strains showed susceptibility against carbapenems (imipenem and meropenem) and tetracycline (minocycline).

**Conclusion:**

RT-PCR and Vitek 2 compact showed a high level of accuracy in the detection of *S*. Typhi. Multidrug resistance was observed, which is an alert for the reduction of antibiotic consumption.

## Introduction

Enteric fever is a severe infectious disease caused by the human-restricted agent *Salmonella enterica* subsp. *enterica*. The genus *Salmonella* has over 2500 serotypes classified into two distinct species: *S. enterica* and *Salmonella bongori*. *S. enterica* subsp. *enterica* consists of 1500 serotypes [1]. *S. enterica* subsp. *enterica* serovar Typhi (*S*. Typhi) and *Salmonella* Paratyphi A, B, and C are extremely adapted to the human host, acting as their typical reservoir [2].

The transport of the disease is either waterborne, foodborne, or through significant person-to-person contact [3]. *Salmonella* is one of the world’s most common causes of food poisoning. Such bacteria can infect a wide range of food products such as poultry, dairy products, or meal, including those of animal origin [4]. *Salmonella* can also cause diseases in the farming of poultry and pigs [5]. The economic loss caused by an infected diet is one of the main concerns for *Salmonella* [6]. In Asia and Africa, the disease continues to be extremely severe, with close to 21 million cases and an estimated 220,000 deaths each year. Enteric fever in endemic areas, especially resource limited, is a significant public health concern [3]. An epidemiological study about Iraq stating that thousands of people are dying every year due to the deficiency of fresh water and contamination of rivers by sewage and factories [7].

The high rate of occurrence of typhoid fever was worsened by the rise of antibiotic-resistant *S. enterica* subsp. *enterica*. To minimize the spread of resistant strains, the Centers for Disease Control and Prevention classified drug-resistant *S*. Typhi as a critical problem that needs regular monitoring and prevention [8]. Antibiotic-resistant *S*. Typhi is the greatest challenge worldwide, especially in countries with poor sanitation, medication facilities, and low income [9]. Emerging multidrug resistance has led to an increment in the death rate as antibiotics are no longer effective. A very distressing investigation about multidrug-resistant (MDR) *S*. Typhi in the Middle East and Central Asia reports that Iraq has the highest prevalence of MDR *S*. Typhi followed by Pakistan [10].

The standard technique to identify *Salmonella*, widely applied for 60 years, includes detecting 3-antigenic locations (somatic O and two antigenic flagella H) using a particular antiserum by slide agglutination, the Kauffmann-White-Le Minor scheme. Despite its global use, this technology is time-intensive, not consistently effective, and needs skilled personnel [11]. It is challenging, particularly in children with uncommon symptoms, to distinguish enteric fever from other undifferentiated febrile diseases, including influenza, leptospirosis, dengue, or malaria [12,13]. Widal test is widely used in many endemic countries, although the sensitivity and specificities are poorly defined as the most common diagnostic procedures [12,14]. Tools such as polymerase chain reaction (PCR) have become central in detecting and typing contagious diseases by providing rapidness, sensitivity, and specificity [3,15,16], however, it is unaffected whether patients were consumed antibiotics or not.

It is very essential to have a rapid and accurate detection for *Salmonella* spp. However, traditional techniques such as the Widal test, Enzyme-linked immunosorbent assay, culturing, and biochemical tests are still regularly used to detect *Salmonella* spp. However, these techniques are laborious and inaccurate. In third world countries, such as Iraq, the rate of detection of bacteria causing enteric fever as well as their antibiotics resistance is hard to assess because of lack of monitoring, insufficient reporting, and publication. For the above reasons, the study aimed to isolate and identify *S*. Typhi from typhoid suspected patients and determine their antibacterial susceptibility pattern.

## Materials and Methods

### Ethical approval and Informed consent

Ethical approval was not needed for this study. We have obtained the samples from the Al-Nokhba Diagnostic Centre, collected by trained personnel and with the doctor’s supervision. The objective of the study was described to all the patients in detail, and a signed consent form was collected.

### Study period and location

The samples were collected from January 2020 to June 2020 in Baghdad, Iraq. The samples were processed in the Hematology Unit and Microbiology Unit at Al-Nokhba Diagnostic Centre.

### Bacterial isolates

The study enrolled patients with high fever (>38°C) who approached Al-Nokhba Diagnostic Centre in Baghdad, Iraq. Thirty blood samples were collected from suspected typhoid patients suffering from fever, headache, and stomach pain. All the patients did not receive antibiotics for at least 2 weeks before sample collection. Blood samples were cultured on Salmonella Shigella (SS) agar (HiMedia, India), and Xylose Lysine Deoxycholate (XLD) agar (HiMedia, India). The selected colonies were subjected to further characterization.

### Biochemical tests

All the suspected colonies were biochemically tested by Vitek 2 compact (bioMérieux, France) using Vitek 2 GN card according to the manufacturer’s instructions. Three to five fresh colonies were transferred into two tubes containing 3 mL of normal saline. The suspension was inoculated into the Vitek 2 compact with a Gram-negative identification test. The biochemical tests employed in the present study are mentioned in Table-1.

**Table-1:** The representative of biochemical characterization of 27 strains of *Salmonella* Typhi by Vitek 2 compact GN card.

No.	Biochemical test	Results	No.	Biochemical tests	Results
1.	ala-phe-pro-arylamidase	−	25.	D-glucose	+
2.	H2S production	−	26.	D-mannose	+
3.	Beta-glucosidase	−	27.	Tyrosine arylamidase	−
4.	L-proline arylamidase	−	28.	Citrate(sodium)	−
5.	Saccharose/sucrose	−	29.	Beta-n-acetyl-galactosaminidase	−
6.	L-Lactate alkalinization	+	30.	L-Histidine assimilation	−
7.	Glycine arylamidase	−	31.	Ellman	−
8.	Adonitol	−	32.	D-cellobiose	−
9.	Beta-n-acetyl-glucosaminidase	−	33.	Gamma-Glutamyl-Glucosaminidase	+
10.	D-maltose	+	34.	Beta-xylosidae	−
11.	Lipase	−	35.	Urease	−
12.	D-tagatose	−	36.	Malonate	−
13.	alpha-glucosidase	−	37.	Alpha-galactosidase	−
14.	Ornithine decarboxylase	−	38.	Coumarate	+
15.	Glu-Gly-Arg-Arylamidase	−	39.	L-lactate assimilation	−
16.	L-pyrrolidonyl-arylamidase	−	40.	Beta-galactosidase	−
17.	Glutamyl arylamidase pNA	−	41.	Fermentation/glucose	+
18.	D-Mannitol	+	42.	Beta-alanine arylamidase pNA	−
19.	Palatinose	−	43.	D-sorbitol	+
20.	D-TREHALOSE	+	44.	5-Keto-D-Gluconate	−
21.	Succinate alkalinization	−	45.	Phosphate	+
22.	Lysine decarboxylase	+	46.	Beta-Glucuronidase	−
23.	L-malate assimilation	−	47.	2,4-Diamino-6,7-Diisopropylpteridine resistance	+
24.	L-arabitol	−		

### DNA extraction

The DNAs of the isolates were extracted using Wizard genomic DNA purification kit (Promega, USA) as instructed by the manufacturer. One hundred microliters of DNA from each sample were eluted and stored at −20°C until use for molecular detection.

### Real-time PCR (RT-PCR) assays

*Salmonella enterica* subsp. *enterica* was detected by targeting invasion A (InvA) gene using genesig^®^ Standard Kit (Primerdesign Ltd., UK). All RT-PCR assays were performed according to the manufacturer’s protocol. Positive and negative controls were involved. The positive control was provided with the same kit above, while RNase-/DNase-free water was used as a negative control. All the cycles were performed on StepOnePlus RT-PCR System (Thermo Fisher Scientific, Massachusetts, US).

### Antibiotics susceptibility pattern

The isolates were tested for their susceptibility to antibiotics by Vitek 2 compact (bioMérieux, France) using GN cassette AST-N222 card (bioMérieux, France) according to the manufacturer’s instructions. The turbidity was adjusted to 0.5 as per McFarland standard by DensiCHEK Plus. The suspension was inoculated into the Vitek 2 compact with GN cassettes AST card. According to Vitek 2 compact system special software, interpretation of the results was performed as explained by the manufacturer’s instructions. Following the Clinical Laboratory Standards Institute criteria, the susceptibility data were analyzed [17]. The antibiotics employed in this study were ticarcillin, ticarcillin/clavulanic acid, piperacillin, cefepime, ceftazidime, aztreonam, amikacin, ­gentamicin, tobramycin, ciprofloxacin, trimethoprim/sulfamethoxazole, minocycline, piperacillin/tazobactam, imipenem, and meropenem.

## Results

**Figure-1:**
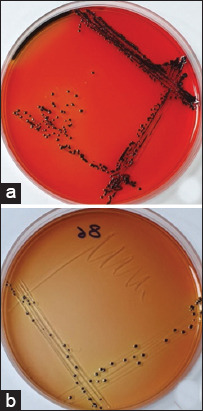
Representative culture media for *Salmonella* Typhi strains on: (a) XLD agar; (b) SS agar.

**Figure-2:**
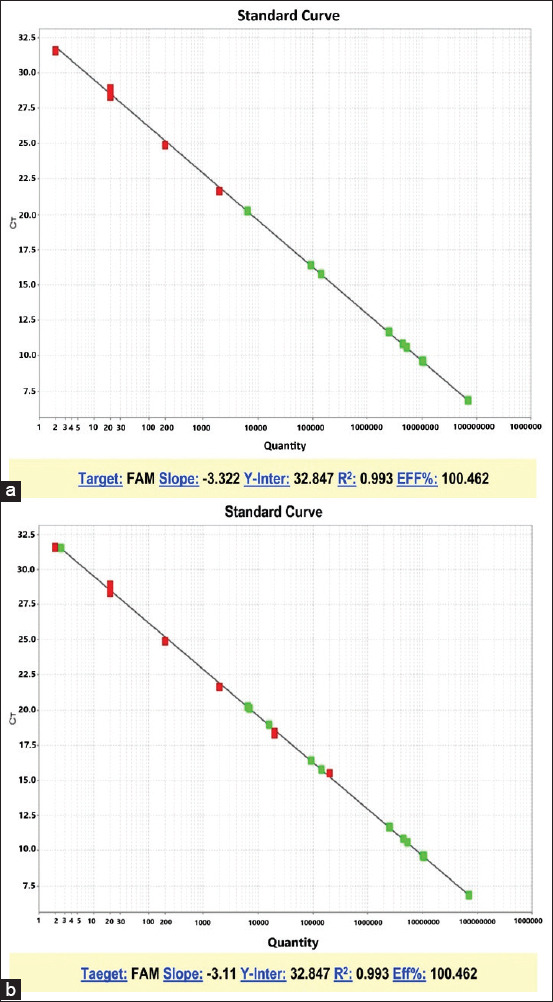
(a and b) The representative standard curve of real-time polymerase chain reaction of *Salmonella* Typhi strains.

Thirty blood samples acquired from 30 suspected typhoid patients were involved in this study. The colony of *Salmonella* was shown as black in color on SS agar and XLD agar due to the production of H_2_S (Figure-1). Of the sample size, 27 isolates were recognized as *S*. Typhi based on biochemical tests (Table-1). In contrast, 29 isolates were identified as *S*. Typhi using RT-PCR (Figure-2).

The antibiotics susceptibility pattern of *S*. Typhi isolates is demonstrated in Table-2. Among the tested antibiotics, the strains of *S*. Typhi shown resistance to 12 antibiotics belong to seven classes. Of them, all *S*. Typhi strains were resistant to penicillins (ticarcillin and piperacillin), cephalosporins 4^th^ G (cefepime), and monobactam (aztreonam). However, a high resistance rate was recorded against other antibiotics. Of the resistant antibiotics, ticarcillin and piperacillin were demonstrated the highest resistance (MIC≥128). All the strains of *S*. Typhi showed susceptibility against carbapenems (imipenem and meropenem) and tetracycline (minocycline). The data revealed that the strains of *S*. Typhi showed MDR.

**Table-2:** Antibiotics susceptibility pattern against *Salmonella* Typhi isolates.

Classes of antibiotics	Antibiotics	*Salmonella* Typhi (n=29)		MIC

		Sensitive (%)	Resistance (%)	
Penicillins	Ticarcillin		29 (100%)	≥128
	Ticarcillin/clavulanic acid	2 (6.9%)	27 (93%)	64
	Piperacillin		29 (100%)	≥128
	Piperacillin/tazobactam	28 (96.5%)	1 (3.4%)	≤4
Cephalosporins 4^th^ generation	Cefepime		29 (100%)	≥64
Cephalosporins 3^rd^ generation	Ceftazidime	1 (3.4%)	28 (96.5%)	≥64
Fluoroquinolone	Ciprofloxacin	1 (3.4%)	28 (96.5%)	0.5
Monobactam	Aztreonam		29 (100%)	≥64
Aminoglycosides	Amikacin	4 (13.7%)	25 (86.2%)	≤2
	Gentamicin	1 (3.4%)	28 (96.5%)	≤1
	Tobramycin	2 (6.9%)	27 (93%)	≤1
Sulfonamides	Trimethoprim/sulfamethoxazole	25 (86.2%)	4 (13.7%)	≤20
Tetracycline	Minocycline	29 (100%)		≤1
Carbapenems	Imipenem	29 (100%)		≤0.25
	Meropenem	29 (100%)		≤0.25

## Discussion

Recently, a massive number of research reports have been done to approve different tools for the identification of *Salmonella* spp. As the world is fighting antibiotic resistance, completing this goal requires a correct, quick, and early diagnosis. Febrile diseases like typhoid fever are among the most prevalent infections in Iraq. However, limited reports for determining the antibacterial susceptibility testing in Iraq were published. Therefore, this project was initiated to detect clinical strains of *S*. Typhi and determine their antibiotics susceptibility pattern.

On SS agar and XLD agar, black color colonies were considered as suspected colonies of *S*. Typhi, due to the production of H_2_S (Figure-1). However, other organisms such as *Proteus* spp. and *Citrobacter* spp. may produce H_2_S, which forms black color colonies on XLD [18]. Hence, the identification based on culture medium is not accurate, which is in agreement with the previous report [19].

Among the sample size, 27 isolates were identified as *S*. Typhi based on biochemical tests (Table-1). The identification based on biochemical tests using Vitek 2 compact was well recommended for Gram-negative bacteria due to its accuracy [20,21]. Furthermore, 29 isolates were confirmed as *S*. Typhi based on RT-PCR. (Figure-2). The identification with Vitek 2 compact exhibited a high level of agreement with the results of RT-PCR. The same level of accuracy was observed in the previous reports [20,22]. In a recent study, the identification of *S. enterica* serovar Gaminara by RT-PCR was considered rapid and less expensive compared with conventional methods [23]. The current data are in accordance with a report presented by Gand *et al*. [24], stating that RT-PCR gives accurate detection of *S. enterica* and saves time with high efficiency, making it an ideal method of diagnosis.

The present study revealed MDR against *S*. Typhi isolated from patients in Baghdad, Iraq (Table-2). These findings are consistent with what was reported earlier in Kenya, where 68% of *S*. Typhi were MDR [25]. However, this contrasts with a study conducted in India that reported a small proportion of *S*. Typhi as MDR [26]. Whereas in Nepal, no MDR has been noted [27]. The high prevalence of MDR is perhaps because of the extensive use of antibiotics without consulting doctors, which is very common in developing countries [28], particularly in Iraq.

Among the resistant antibiotics, 100% of *S*. Typhi strains were resistant to penicillins (ticarcillin and piperacillin) which displayed the highest frequency of resistance (MIC≥128) (Table-2). These two antibiotics are broad-spectrum beta-lactam. Similar outcomes were found in *Salmonella* spp., isolated from poultry [29,30]. Different studies reported the resistance of *Salmonella* spp. to ticarcillin [31,32]. The current result is contrary to a case report conducted by Perera *et al*. [33] who showed a sensitivity o*f S*. Typhi against piperacillin.

Piperacillin is regularly used in combination with tazobactam (piperacillin/tazobactam), which improves piperacillin efficiency by inhibiting the enzymes of beta-lactamases in different species. The present study revealed that 96.5% of *S*. Typhi strains were sensitive to piperacillin/tazobactam (MIC≤4) (Table-2). This finding is similar to the results of Perera *et al*. [33]. Moreover, Lowman stated that piperacillin/tazobactam remains a valuable antibiotic against Gram-negative bacteria [34]. Ticarcillin is widely used in combination with clavulanic acid (ticarcillin/clavulanic acid) to treat Gram-negative bacteria [32]. However, 93% of *S*. Typhi strains showed less resistance to ticarcillin/clavulanic acid (MIC 64). This contradicts the findings reported earlier, which demonstrated ticarcillin/clavulanic acid activity against different Gram-negative bacteria [35].

Among the isolated strains, 96.5-100% of *S*. Typhi strains displayed moderate resistance (MIC≥64) to cephalosporins 3^rd^ G (ceftazidime), cephalosporins 4^th^ G (cefepime), and monobactam (aztreonam) (Table-2). This is not in accordance with the study of Yang *et al*. [36] that reported the sensitivity of *Salmonella* spp. to ceftazidime and cefepime. The present study is comparable to the study reported earlier, indicating the resistance of *S. enterica* serotype Typhimurium to aztreonam [37]. Recent studies reported that the combination of aztreonam and ceftazidime-avibactam was improved the medication against Gram-negative bacteria [38-40].

Amikacin, tobramycin, and gentamicin belong to a group of antibiotics called aminoglycosides. In the present investigation, 86.2-96.5% of *S*. Typhi strains were resistant to all antibiotics of aminoglycosides (Table-2). Lo *et al*. [41] reported the low activity of aminoglycosides against intracellular *S. enterica* serovar Typhimurium. Furthermore, 86.2% of *S*. Typhi strains were found to be resistant to amikacin (Table-2). Studies conducted in India and Ethiopia demonstrated the susceptibility of *S*. Typhi to amikacin [42,43], which is not in agreement with our results. *S*. Typhi strains (96.5%) were found to be resistant to gentamicin (MIC≤1) (Table-2), which is not in accordance with previous reports that showed a susceptibility of *S*. Typhi to gentamicin [25,43]. Moreover, recent data reported that Gram-negative bacteria were sensitive to gentamicin [40]. The present study specified the resistance of 93% of *S*. Typhi strains to tobramycin (Table-2). This is strongly contradicted with a study conducted in Baghdad, Iraq, which demonstrated the sensitivity of 80% of *S*. Typhi strains to tobramycin [44]. This is a clear indication of the resistance increasing of *S*. Typhi to tobramycin.

Moreover, 96.5% of strains of *S*. Typhi have also exhibited resistance to ciprofloxacin (Table-2). The previous studies reported increasing numbers of resistance of *S*. Typhi to ciprofloxacin [25,45,46]. However, another study in Ethiopia revealed the susceptibility of *S*. Typhi to ciprofloxacin [43], which is contrary to the current research. As per the literature review, ciprofloxacin is a substitute antibiotic for the treatment of MDR-*Salmonella* spp. Reduced ciprofloxacin sensitivity has a significant risk to the failure of typhoid fever medication, particularly in developing countries. Among the Middle East countries, Iraq was shown the highest sensitivity reduction in ciprofloxacin [10], which is in accordance with the present study. Moreover, the current study is comparable to a study from Baghdad, where *S*. Typhi was resistant to ciprofloxacin [44].

On the other hand, the study documented the sensitivity of *S*. Typhi against imipenem, meropenem, minocycline, and trimethoprim/sulfamethoxazole (Table-2). In contrast, a study in Indonesia reported resistance of *S*. Typhi to imipenem, meropenem, and trimethoprim/sulfamethoxazole [47], while a study in Nepal displayed a sensitivity of *S*. Typhi to trimethoprim/sulfamethoxazole [27]. Whereas in Pakistan, a high resistance rate of *S*. Typhi to trimethoprim/sulfamethoxazole was observed [48]. Minocycline has been an effective anti-MDR antibacterial for over the past 30 years [49]. In contrast to our results, a study from South Korea reported resistance of 30 strains of *Salmonella* spp. to minocycline [50].

According to the present findings, *S*. Typhi strains were shown the highest resistance against ticarcillin, piperacillin, and cefepime. Therefore, we strongly advise not to use these antibiotics for typhoid treatment. On the other hand, imipenem and meropenem were presented the highest sensitivity.

## Conclusion

The study concluded that the use of Vitek 2 compact and RT-PCR in detecting *S*. Typhi is giving accurate results which are very important for the diagnosis of typhoid. According to the above findings, we observed increasing numbers of antibiotic-resistant *S*. Typhi, which is required to raise the level of awareness of consuming antibiotics. Continuous surveillance of the antibacterial sensitivity testing and a reasonable dosage of antibiotics should be maintained to avoid the outbreak of multidrug resistance.

## Authors’ Contributions

HAS: Wrote and corrected the manuscript as well as analyzed the data. HAS, AMA, and MNF: Did the laboratory work, including Vitek 2 compact and real-time PCR. ZMR: Collected the samples and managed the data as well as laboratory work such as Vitek 2 compact antibiotics susceptibility testing. All authors have read and approved the final manuscript.
